# Sexual Function and Sexual Quality of Life in Premenopausal Women with Controlled Type 1 and 2 Diabetes—Preliminary Study

**DOI:** 10.3390/ijerph18052536

**Published:** 2021-03-04

**Authors:** Ewelina Bąk, Agnieszka Młynarska, Danuta Sternal, Monika Kadłubowska, Ewa Marcisz-Dyla, Czesław Marcisz

**Affiliations:** 1 Faculty of Health Sciences, University of Bielsko-Biala, 43-309 Bielsko-Biala, Poland; dsternal@ath.bielsko.pl (D.S.); mkadlubowska@ath.bielsko.pl (M.K.); 2 Department of Gerontology and Geriatric Nursing, Faculty of Health Sciences, Medical University of Silesia, 40-635 Katowice, Poland; amlynarska@sum.edu.pl (A.M.); klinwewtychy@poczta.onet.pl (C.M.); 3 Faculty of Management, Psychology, Katowice Business University, 40-659 Katowice, Poland; eva.marcisz@gmail.com

**Keywords:** sexual function, type 1 and 2 diabetes, menstrual cycle

## Abstract

Sexual dysfunction is more common in women with diabetes than in women without diabetes. The aim of the study was to determine sexual function and the level of the quality of sex life in premenopausal women with controlled, uncomplicated type 1 and type 2 diabetes taking into account the stages of the menstrual cycle and mood level. The study included 163 women with type 1 and type 2 diabetes and 115 controls without diabetes. Questionnaire studies were conducted using the following surveys: Demographic and Clinical Data Survey, Female Sexual Function Index, Sexual Quality of Life—Female, and Beck Depression Inventory. Both phases of the menstrual cycle—follicular and luteal—were included. It was shown that, in women with type 1 diabetes, sexual function decreased during the luteal phase in comparison with the follicular phase (*p* < 0.001). In the women with type 2 diabetes and in the controls, sexual function was comparable during both phases of the cycle (*p* > 0.05). In the women with uncomplicated controlled type 1 diabetes, sexual function and the sexual and relationship satisfaction changed depending on the phase of the menstrual cycle with a decrease during the luteal phase. Sexual function and the quality of the sex life of premenopausal women with controlled type 2 diabetes were comparable during both the follicular and the luteal phases. Sexual function in menstruating women with controlled type 2 diabetes decreased with age and a worsening mood.

## 1. Introduction

Diabetes is a common chronic disease that is classified as a civilization disease. A distinction is made between type 1 diabetes (T1DM), which is associated with an insulin deficiency, and type 2 diabetes (T2DM), which primarily results from insulin resistance. This disease leads to many chronic complications, which include sexual dysfunction in both men and women [1,2,3]. Men with diabetes mainly have erectile dysfunction [1,2,3]. Women with diabetes reported problems connected with desire, excitement, orgasm, satisfaction, and vaginal pain and hydration significantly more often than those who did not have diabetes [4,5]. Patients with both type 1 and type 2 diabetes experienced a decline in the quality of sex life, which is an important though often overlooked component of human wellbeing [5,6]. Research shows that 25–71% of women with T1DM [2,7,8,9,10,11,12] and 25–68% of women with type 2 diabetes [1,7,13,14] suffer from female sexual dysfunction (FSD). It has been shown that sexual disorders in women with T1DM and in those with T2DM demonstrate a positive correlation with the occurrence of depression [1,2], and, in diabetic women during menopause, a depressed mood was also shown to be an independent indicator of FSD [15].

The assessment of the sexual functions of menstruating and fertile diabetic women during the follicular and luteal phases seems to be interesting and important from the point of view of clinical sexology. According to the literature review, only one study has been devoted to this issue so far. It was published in 2006 and included women with T1DM. It was shown that these women had decreased sexual function and increased sexual distress compared with control subjects during the luteal, but not the follicular, phase of the menstrual cycle [16].

Due to the small amount of research and the complexity of sexual problems, as well as cultural and methodological difficulties, women’s sexual dysfunction still remains a silent complication. Sexuality, its multidimensionality, and individual perception make determining the predictive factors of sexual disorders in women quite difficult and not very accurate [10,17,18,19]. The empirical limitations in assessing the sexuality of women with diabetes justify the legitimacy of undertaking studies covering their sexual activities in relation to the quality of sex life.

The aim of the study was to determine the level of sexual activity and the level of the quality of sex life in premenopausal women with controlled, uncomplicated type 1 and type 2 diabetes taking into account both stages of the menstrual cycle and mood level.

## 2. Material and Methods

### 2.1. Population

Cohort studies were conducted in 206 patients with diabetes (91 with T1DM and 115 with T2DM) and 133 nondiabetic women in the control groups. Because some of the women refused to participate in the study or because their questionnaires were incomplete, only 70 women with T1DM aged 22.9 ± 2.0 (mean ± SD) years (Group I) and 93 women with T2DM aged 39.1 ± 6.5 years were qualified for the study (Group II), in addition to 58 controls (Group CI)—corresponding to the age of Group I and 57 controls (Group CII))—corresponding to the age of Group II. Taking into account the phase of the menstrual cycle, Group I included 32 patients in the follicular phase (Group IA) and 38 patients in the luteal phase (Group IB), and Group II included 39 patients in the follicular phase (Group IIA) and 54 patients in the luteal phase (Group IIA) IIB), while, in the CI Group, there were 31 women in the follicular phase (CIA Group) and 27 women in the luteal phase (CIB Group) and, in the CII Group, there were 32 women in the follicular phase (CIIA Group) and 25 women in the luteal phase (CIIB Group).

The criteria for inclusion in the study were age from 18 to 45 years, duration of diabetes at least 1 year after diagnosis, glycated hemoglobin (HbA1c) <7%, sexual activity with at least two sessions of intercourse a week, regular menstruation, persistence in heterosexual relationships, and giving informed consent to participate in the research. The study excluded people with cancer, endocrine gland diseases, alcoholism, inflammation within the last 4 months, and the use of immunosuppressive and anti-inflammatory drugs, glucocorticoids, psychotropic drugs, or oral contraceptives. The T1DM patients were treated with insulin and followed the recommended diet. However, for the women with T2DM, the treatment consisted of compliance with the diet and the use of oral antidiabetic drugs from the sulfonylurea and biguanidine groups, although several patients were also given insulin. All of the individuals that were included in the study were patients of the Clinic Practice Group of Doctors in Kaczyce Silesian Province. Patients with T1DM and T2DM were recruited for participation when they visited their doctor to monitor their health in terms of their carbohydrate balance and blood pressure. The women from the control groups did not have any chronic diseases and did not take any medication. The participants were recruited by their doctor during routine periodic medical examinations. All subjects were informed about the study protocol. Participants voluntarily agreed to participate in the study and all signed informed consent. The research was conducted from June 2017 until December 2019.

### 2.2. Methodology

In all examined women, fasting blood glucose level was determined. The HbA1c level was determined in examined patients only. Systolic and diastolic blood pressure was measured and body mass index (BMI) was calculated. Subsequently, questionnaire studies were conducted using the following surveys: Demographic and Clinical Data Survey, Female Sexual Function Index (FSFI), Sexual Quality of Life—Female (SQoL-F), and Beck Depression Inventory (BDI). It was recommended that the answers to the questions in FSFI, SQoL-F, and BDI cover the last 7 days. The questionnaire studies were conducted in the second half of the follicular or luteal phase. The questionnaires were self-administered and fulfilled independently by each participant included in the study at the clinic without any possible influence from medical staff.

#### 2.2.1. Demographic and Clinical Data Survey

The survey included parameters such as age, education, place of residence, marital status, professional activity, stimulants being used, duration of diabetes, comorbidities, and medications being taken.

#### 2.2.2. Female Sexual Function Index (FSFI)

The FSFI questionnaire, which was developed by Rosen et al. [20], consists of 19 questions and produces a multidimensional assessment of a woman’s sexual functions. The index has been standardized and adapted (in many language versions, including Polish) in order to differentiate sexual dysfunction in women aged 18–70 according to the current classifications and recommendations of the scientific societies. It has a documented sensitivity, reliability, internal consistency, and reliability, as well as a stability and repeatability of the results. The questions in the questionnaire are grouped into six domains: desire, arousal, lubrication, orgasm, satisfaction, and pain. The final results are obtained separately for each domain and are added up (total FSFI). To assess the individual domains, the number of points that can be obtained ranges from 0 to 6 for excitement, lubrication, orgasm, and pain-related disorders, 0.8–6.0 for sexual satisfaction, and 1.2 to 6.0 for the function of desire. In the global assessment, the range of points that can be obtained is between 2 and 36.

#### 2.2.3. Sexual Quality of Life in Women (SQoL-F)

The SQoL-F questionnaire was developed by Symonds et al. [21]. It consists of 18 questions, and each item is rated on a six-point response (completely agree to completely disagree). The response categories can be scored as either 1 to 6 or 0 to 5 in order to obtain a total of 18–108 or 0–90 items. Each item can be scored either 1 to 6 or 0 to 5 with items 1, 5, 9, 13, and 18 being reverse-scored items. The calculation is divided into four factors: factor 1 (psychosexual feelings), which includes seven items (items 2, 3, 7, 8, 10, 16, and 17), factor 2 (sexual and relationship satisfaction), which includes five items (items 1, 5, 9, 13 and 18), factor 3 (self-worthlessness), which includes three items (items 4, 6, and 15), and factor 4 (sexual repression), which includes three items (items 11, 12, and 14). These are used to assess the impact of sexual disorders on the quality of life in three aspects—emotional wellbeing, self-esteem, and relationship. The index has no cutoff score and a high total score for the index indicates that the sexual quality of life is quite good.

#### 2.2.4. Beck Depression Inventory (BDI)

The BDI is a 21-point screening questionnaire that is used to assess the severity of mood disorder (depression) symptoms. The scale consists of 21 questions that score from 0 to 3 points. The results that can be obtained in the BDI range from 0 to 63. The BDI is a questionnaire that has been standardized and validated to Polish conditions, which is repeatedly used in studies to assess mood disorders [22]. Before starting the study, each individual was informed about the purpose of the study. During the medical visit, the questionnaires were completed independently and anonymously.

### 2.3. Statistical Analysis

Statistical calculations were done using the Statistica 13 software. Normality tests were performed using the Shapiro–Wilk W-test. The results are presented for the data with a normal distribution using the mean and standard deviation, while those for the data without a normal distribution are presented for the median and first and third quartiles. The qualitative data are presented in terms of numbers and percentages. In order to determine the homogeneity of the groups, the Student’s *t*-test was used for the data with normal distribution, while, for the data without a normal distribution, the Mann–Whitney U test was used, and, for the qualitative data, a series of χ^2^ tests were used (the χ^2^ test with the highest reliability, the χ^2^ test with the Yates correction, or the V-square test). When examining the impact of the different factors on the level of the quality of a participant’s sex life and sexual function, the multiple stepwise regression method was used, which resulted in linear relationships being obtained (the result was the equation of a straight line with many parameters). To compute the effect of two covariate factors such as group and phase of the menstrual cycle on FSFI, SQoL-F, and BDI, the MANOVA.RM (repeated measures) package was used, where the available function provides the Wald-type statistic. This package also contains the simCI() function which allows multivariate post hoc comparisons. Tukey’s honestly significant difference test was used for multiple comparisons in this article. The study assumed a 0.05% significance level.

## 3. Results

It was shown that, in the women with T1DM and the controls, the values of the sociodemographic parameters and systolic and diastolic blood pressure, which were determined during the follicular and luteal phases of the menstrual cycle, proved to be statistically comparable (*p* > 0.05; Table 1). The duration of diabetes in the patients during the follicular and luteal phases did not differ significantly. The fasting glucose and HbA1c levels were slightly significantly higher (*p* < 0.05) in the patients during the luteal phase than during the follicular phase (Table 1).

All of the examined patients were treated with insulin, followed the prescribed diet, and did not use any oral medications. In these patients, there were no clinically apparent chronic diabetes complications such as retinopathy, nephropathy, polyneuropathy, or diabetic foot, or their associated diseases.

In the women with T2DM and the controls, the values of the sociodemographic parameters and systolic and diastolic blood pressure, which were determined during the follicular and luteal phases of the menstrual cycle, proved to be statistically comparable (*p* > 0.05; Table 2). The duration of diabetes, the occurrence of concomitant diseases, the use of drugs, fasting glucose, and HbA1c in the patients during the luteal and follicular phases did not differ significantly (*p* > 0.05; Table 2).

Using MANOVA in the patients with T1DM, it was found that the values of all of the FSFI domains and the score of factor 2 (sexual and relationship satisfaction) of the SQoL-F were significantly lower during the luteal phase than in the follicular phase (*p* < 0.01–0.001; Table 3 and Table 4). In the control groups, the tested values of the FSFI domains and the SQoL-F factors during the menstrual cycle phases did not differ significantly (*p* > 0.05; Table 4). The BDI values were comparable during both of the studied phases of the menstrual cycle in both the patients with T1DM and the controls (*p* > 0.05) (Table 3 and Table 4).

Using a MANOVA in the patients with T2DM and the controls, there were no significant differences between the values in the domains of FSFI, the SQoL-F factors, and the BDI on the phases of the menstrual cycle (*p* > 0.05; Table 5 and Table 6).

Cronbach’s α coefficients in examined groups for the used FSFI, SQoL-F, and BDI questionnaires were 0.938–0.972, 0.735–0.784, and 0.899–0.939, respectively, suggesting that they have relatively high internal consistency.

A multiple regression analysis showed that the total FSFI score in the women with T1DM was significantly lower during the luteal phase (*p* < 0.001). The model was statistically significant and explained 42.9% of the observed variability in the dependent variable (*p* < 0.0001). In the patients with T2DM, the total FSFI score decreased with age (*p* = 0.002) and decreased as their mood deteriorated (*p* < 0.001). The model was statistically significant and explained 35.8% of the observed variability in the dependent variable (*p* < 0.0001; Table 7).

A multiple regression analysis showed that the quality of the sex life (SQoL-F) of the women with T1DM was significantly lower during the luteal phase (*p* < 0.001), decreased with age (*p* = 0.004), and increased in patients with higher total FSFI (*p* < 0.001). The model was statistically significant and explained 38.9% of the observed variability in the dependent variable (*p* < 0.0001; Table 8). In turn, the patients with T2DM had a significantly higher SQoL-F level with their higher total FSFI (*p* < 0.001). The model was statistically significant and explained 44.3% of the observed variability in the dependent variable (*p* < 0.0001; Table 8).

## 4. Discussion

This study included sexually active regularly menstruating females who had controlled T1DM and T2DM and the corresponding age-related sexually active nondiabetic women. The participants with T1DM and T2DM are discussed separately, mainly due to the differences in terms of age, the occurrence of concomitant diseases, and the method of treatment, as well as the other etiopathomechanisms of the disease.

It was demonstrated that, in the patients with uncomplicated T1DM, the level of sexual activity in all of the subjects using the FSFI domains (desire, arousal, lubrication, orgasm, satisfaction, and pain) was lower during the luteal phase than it was during the follicular phase (*p* < 0.001). In the nondiabetic control group, the results of sexual function that were obtained during both phases of the menstrual cycle were comparable. In the available literature, only one publication was found that concerned the sexuality test in patients with T1DM, which took into account the phases of the menstrual cycle [16]. The authors of the study proved that, in these patients, as in their own studies, there was a decrease in sexual function as determined using the FSFI only during the luteal phase. Without taking into account the phase of the menstrual cycle, other authors also described a higher incidence of decreased sexual function in premenopausal women with T1DM. Dimitropoulos et al. [8] showed that FSD occurred in every fourth patient with uncomplicated T1DM, which was three times more often than in nondiabetic women. In a study of menstruating women with T1DM, sexual dysfunction was observed in 27% of women, including 22% with uncomplicated diabetes and 33% with complications [23]. Other studies in menstruating women showed a significant reduction in sexual function in 29–44% of women with T1DM and 5–13% of comparable women without diabetes [2,7,11,12]. It is emphasized that, in patients with T1DM, there were mainly desire and arousal disorders [2,8].

The multifactorial regression analysis that we used showed that the level of sexual function in women with T1DM was only determined by the menstrual phase and was not related to age and mood level. Similar results were obtained by Salonia et al. [16], demonstrating that, in menstruating women with T1DM, decreased sexual function occurs independently of mood deflections. In turn, other studies have shown relationships between sexual dysfunction in menstruating women with both T1DM and depression [2,8,23]. Enzlin et al. [10] even suggested that depression is an essential predictor of sexual dysfunction in women with T1DM.

Our research showed that the level of the sexual and relationship satisfaction (factor 2 of SQoL-F) of the patients with T1DM was lower during the luteal phase than during the follicular phase. The demonstrated reduced sexual and relationship satisfaction in the luteal phase compared to the follicular phase may be related to the observed increase in discomfort or pain at sexual penetration during the luteal phase. In addition, the lower factor 2 value of SQoL-F in the luteal phase in T1DM coincides with the satisfaction domain value in the FSFI. A multiple regression analysis showed that total SQoL-F of these women was lower during the luteal phase, decreased with age, and increased in patients with higher total FSFI score.

The changes in sexual function and the quality of sexual life seemed to depend mainly on diabetes itself as an independent risk factor for sexual dysfunction because, in the examined group of women, there were no cases with complications of this disease or any other accompanying diseases, and no drugs other than insulin were used. Similarly, Flotynska et al. [11] suggested that T1DM was independently associated with the presence of impaired sexual function. Other studies showed that the complications had an adverse effect on the sexual function of women with T1DM diabetes [2,23]. In our study, the occurrence of depression symptoms, which were determined using the BDI, was comparable during both phases of the menstrual cycle; therefore, the observed mood level did not determine whether the diversity of sexual activity or the quality of sexual life was dependent on the phase of the menstrual cycle.

After considering the menstrual phase, the patients with controlled, uncomplicated T2DM had comparable levels of sexual function in all of the FSFI domains that were tested during the follicular and luteal phases. In the available literature, no work was found that involved studies of sexual function in patients with T2DM during the phases of the menstrual cycle. In this study, we showed that patients with T2DM had decreased sexual function in older women and with a lower level of the mood.

Consistent with the level of sexual function, the quality of sexual life was also not different in patients with T2DM depending on the phase of the menstrual cycle. It was found that the level of the quality of the sex life of these patients had a positive correlation with total FSFI score. Celik et al. [5] also showed that women with T2DM mainly showed a significantly positive correlation between the total FSFI score and the total SQoL-F score.

On the basis of our studies, it is not possible to explain the observed differences in sexual function levels in women with T1DM and T2DM depending on the cycle phase. It can only be assumed that hormonal factors, mainly ovarian steroids which modulate a woman’s sexual activity [24], and nonhormonal factors, such as psychological and even sociodemographic conditions, were involved in this mechanism. Further in-depth research is needed to clarify this issue.

Our research had some limitations. Firstly, sexual function was not tested in the same women, both in the follicular and in the luteal phases, which resulted, among other reasons, from the lack of consent to conduct repeated tests; this did not allow observing longitudinal changes in sexual function and in SQoL-F in women with T1DM and T2DM and in the control groups. Secondly, in the studied women, the hormonal parameters such as estradiol, follicle-stimulating hormone, luteinizing hormone, progesterone, prolactin, and androgens in the blood serum in the luteal and follicular phases were not determined, which could shed light on the mechanism explaining the difference in the level of sexual function depending on the cycle phase in T1DM and the absence of that difference in T2DM. Thirdly, the number of women in the individual groups was relatively small; this was related to the difficulty in selecting homogeneous groups in terms of sexual activity, regular menstruation and its phase, the absence of diabetes complications, and evenness of the disease and age (for our sample of diabetics, the maximum error was 10% and the confidence level was 95%). Fourthly, the patients were recruited only from the diabetes outpatient clinic, which means that they are not fully representative of the entire population of women with diabetes. Fifthly, partner relationships and mutual emotional and sexual expressions were not studied, which could have been important in assessing the sexuality of the women that were surveyed.

## 5. Conclusions

In regularly menstruating women with uncomplicated, controlled T1DM, sexual function and sexual and relationship satisfaction changed depending on the phase of the menstrual cycle with a decrease during the luteal phase. Sexual function and the quality of sex life of premenopausal women with controlled T2DM were comparable during both the follicular and the luteal phases. Sexual function in the menstruating women with controlled T2DM decreased with age and a worsening mood.

### Ethical Approval

The research was approved by the Bioethical Commission of the Beskid Medical Chamber in Bielsko-Biała at the meeting on 16 February 2017 (consent No. 2017/02/16/8).

All of the procedures that were performed in the study involving human participants were in accordance with the ethical standards of the committee and with the 1964 Declaration of Helsinki.

## Figures and Tables

**Table 1:** General characteristics of the patients with type 1 diabetes and the controls.

Parameter		Type 1 Diabetes			Controls		
		Follicular Phase (Group IA) (*n* = 32)	Luteal Phase (Group IB) (*n* = 38)	*p*	Follicular Phase (Group CIA) (*n* = 31)	Luteal Phase (Group CIB) (*n* = 27)	*p*
Age (years) (mean ± SD)		22.6 ± 2.2	23.2 ± 1.8	0.249	23.9 ± 3.5	23.1 ± 2.6	0.355
BMI (kg/m^2^) (mean ± SD)		23.6 ± 3.2	24.2 ± 3.1	0.408	22.9 ± 4.2	24.8 ± 4.0	0.491
Education (*n*)	Primary/vocational	9	11	0.680	2	1	0.764
	Preuniversity	15	13		18	18	
	University/higher vocational	8	14		11	8	
Place of Residence (*n*)	Rural area	18	25	0.414	10	7	0.600 ^2^
	Urban area	14	13		21	20	
Marital Status (*n*)	Partnership	4	11	0.315	23	20	0.992 ^2^
	Married	27	26		8	7	
	Widowed	1	1		0	0	
Currently Working/Not Working (*n*)		28/4	37/1	0.205	19/12	20/7	0.362
Smoking: Never/Past/Present (*n*)		16/7/9	20/9/9	0.761 ^1^	20/5/6	18/5/4	0.914 ^1^
Alcohol: Drinking/Not Drinking (*n*)		20/12	21/17	0.540	20/11	12/15	0.124
Duration of Diabetes (years)		5.2 (3–7)	5.3 (3–7)	0.837 ^3^	-	-	-
Systolic Blood Pressure (mm Hg)		121 (119–125)	124.5 (120–125)	0.164	116 (110–120)	115 (110–120)	0.942
Diastolic Blood Pressure (mm Hg)		69 (65–70)	70 (65–71)	0.484	65 (60–70)	70 (60–72)	0.581
Fasting Glucose (mg/dL)		118 (100–119.5)	120 (113–128)	0.012 ^3^	86 (83–89)	80 (78–87)	0.043 ^3^
Post-meal Glucose (120 min) (mg/dL)		138 (135–140)	140 (133–145)	0.199 ^3^			
HbA1c (%)		6.1 (6.0–6.2)	6.2 (6.0–6.3)	0.013 ^3^			

Data are presented as medians (first and third quartiles). Notes: ^1^ χ^2^ test with Yates correction, ^2^ V-squared test, ^3^ Mann–Whitney U test; with no designations—χ^2^ test for the qualitative data and Student’s *t*-test for the quantitative data. Abbreviations: *n*—number, *p*—statistical significance of the differences, BMI—body mass index, HbA1c—glycated hemoglobin.

**Table 2:** General characteristics of the patients with type 2 diabetes and the controls.

Parameter		Type 2 Diabetes			Controls		
		Follicular Phase (Group IIA) (*n* = 39)	Luteal Phase (Group IIB) (*n* = 54)	*p*	Follicular Phase (Group CIIA) (*n* = 32)	Luteal Phase (Group CIIB) (*n* = 25)	*p*
Age (years) (mean ± SD)		40.4 ± 6.6	38.2 ± 6.4	0.110	40 ± 5.2	40.3 ± 4.9	0.855
BMI (kg/m^2^) (mean ± SD)		26.3 ± 4.2	24.8 ± 4	0.071	26 ± 4.1	25.3 ± 3.5	0.521
Education (*n*)	Primary/vocational	8	14	0.636	3	2	0.387
	Preuniversity	12	15		8	4	
	University/higher vocational	19	25		21	19	
Place of Residence (*n*)	Rural area	15	23	0.689	15	14	0.494
	Urban area	24	31		17	11	
Marital Status (*n*)	Partnership	10	15	0.455	7	5	0.930
	Married	26	36		25	20	
	Widowed	3	3		0	0	
Currently Working/Not Working (*n*)		32/7	50/4	0.219 ^1^	32/0	25/0	1
Smoking: Never/Past/Present (*n*)		27/6/6	30/12/12	0.777 ^3^	23/3/6	18/2/5	0.906 ^2^
Alcohol: Drinking/Not Drinking (*n*)		20/19	19/35	0.121	9/23	5/20	0.483 ^2^
Duration of Diabetes (years)		5 (2–8.5)	4.5 (2–8)	0.744 ^3^			
Drugs: Oral Antidiabetic/Insulin (*n*)		39/8	54/3	0.687 ^2^			
Comorbidities: Hypertension/Coronary Heart Disease (*n*)		6/1	7/0	0.741 ^2^	6/0	1/0	0.202 ^1^
Systolic Blood pressure (mm Hg)		123 (120–130)	123.5 (120–130)	0.905 ^3^	120 (117–125)	120 (110–121)	0.098 ^3^
Diastolic blood pressure (mm Hg)		70 (69–80)	70 (65–80)	0.182 ^3^	65.5 (61.5–72.5)	67 (60–75)	0.801 ^3^
Fasting glucose (mg/dL)		110 (99–120)	110 (95–120)	0.301 ^3^	86 (80–88.5)	86 (80–87)	0.764 ^3^
Post-meal glucose (120 min) (mg/dL)		140 (130–160)	133.5 (130–140)	0.088 ^3^			
HbA1c (%)		6.9 (6.1–7.1)	6.2 (6.1–7)	0.117 ^3^			

Data are medians (first and third quartiles) Notes: with no designations—χ^2^ test for the qualitative data and Student’s *t*-test for the quantitative data; ^1^ χ^2^ test with the Yates correction; ^2^ V-squared test; ^3^ Mann–Whitney U test. Abbreviations: *n*—number, *p*—statistical significance of the differences, BMI—body mass index, HbA1c—glycated hemoglobin.

**Table 3:** The effect of two covariate factors, i.e., group and phase on FSFI, SQoL-F, and BDI with the use of MANOVA.

Type 1 Diabetes	FSFI			SQoL-F			BDI (Score)		
	Test Statistic ^1^	df	*p*	Test Statistic ^1^	df	*p*	Test Statistic ^1^	df	*p*
Group	10.109	6	0.12	31.232	4	<0.001	15.756	1	<0.001
Phase of the menstrual cycle	20.707	6	0.002	37.938	4	<0.001	1.045	1	0.307
Group × phase	31.392	6	<0.001	54.558	4	<0.001	2.083	1	0.149

FSFI—Female Sexual Function Index; SQoL-F—Sexual Quality of Life in Women; BDI—Beck Depression Inventory; df—degrees of freedom; ^1^ value of the Wald-type statistic; *p*—statistical significance of the differences.

**Table 4:** Sexual functions, sexual quality of life, and mood level in fertile women with type 1 diabetes and the controls during the follicular or luteal phases of the menstrual cycle.

Parameter		Type 1 Diabetes			Controls		
		Follicular Phase (Group IA) (*n* = 32)	Luteal Phase (Group IB) (*n* = 38)	*p*	Follicular Phase (Group CIA) (*n* = 31)	Luteal Phase (Group CIB) (*n* = 27)	*p*
**FSFI**	Desire	6.0 (5.4–6.0)	4.2 (3.6–4.8)	<0.01	4.8 (4.8–5.4)	4.8 (4.8–5.4)	1.00
	Arousal	5.6 (5.1–6.0)	4.4 (3.9–4.8)	<0.01	5.1 (5.1–5.7)	5.1 (5.1–5.4)	0.99
	Lubrication	5.7 (5.4–6.0)	4.5 (4.2–5.1)	<0.01	5.4 (5.1–6)	5.7 (5.4–6)	0.96
	Orgasm	5.6 (5.2–5.8)	4.4 (4–4.8)	<0.01	5.2 (4.8–5.6)	5.2 (4.4–5.6)	0.99
	Satisfaction	5.6 (5.2–6.0)	4.4 (4–4.8)	<0.01	5.2 (4.8–5.6)	5.4 (5.2–5.9)	0.82
	Pain	5.6 (5.2–6.0)	4.4 (4–5.2)	<0.01	5.2 (4.8–6)	5.2 (4.8–6)	0.96
	Total FSFI	33.1 (31.55–35.1)	25.85 (24.5–27.4)	<0.01	31.5 (29.8–32.2)	31.9 (30.5–33.4)	0.980
**SQoL-F**	Factor 1	40.5 (33.5–42.0)	38 (36–40)	1.00	36 (31–39)	39 (33–41)	0.96
	Factor 2	27 (25–30)	15 (15–17.5)	<0.01	26 (22–27)	25 (20–27)	0.99
	Factor 3	17 (15–18)	17 (16–18)	1.00	16 (13–18)	16 (14–18)	0.99
	Factor 4	17.5 (14–18)	17 (16–18)	0.85	16 (14–17)	17 (14–18)	0.99
	Total SQoL-F	99 (92–103)	88.5 (77.5–93)	0.10	91 (77–100)	95 (85–100)	0.99
**BDI (score)**		3 (1–4.5)	2 (0–3)	0.96	2 (0–9)	8 (1–14)	0.83

Data are medians (first and third quartiles) Notes: *p*-values (Tukey’s test); FSFI—Female Sexual Function Index; SQoL-F—Sexual Quality of Life in Women; Factor 1—psychosexual feelings; Factor 2—sexual and relationship satisfaction; Factor 3—self-worthlessness; Factor 4—sexual repression; BDI—Beck Depression Inventory; *p*—statistical significance of the differences.

**Table 5:** The effect of two covariate factors, i.e., group and phase, on FSFI, SQoL-F, and BDI with the use of MANOVA.

Type 2 Diabetes	FSFI			SQoL-F			BDI (Score)		
	Test Statistic ^1^	df	*p*	Test Statistic ^1^	df	*p*	Test Statistic ^1^	df	*p*
Group	6.721	6	0.347	2.212	4	0.697	0.065	1	0.799
Phase of the menstrual cycle	4.392	6	0.624	3.679	4	0.451	0.007	1	0.931
Group × phase	4.784	6	0.572	1.979	4	0.74	0.912	1	0.34

FSFI—Female Sexual Function Index; SQoL-F—Sexual Quality of Life in Women; BDI—Beck Depression Inventory; df—degrees of freedom; ^1^ value of the Wald-type statistic; *p*—statistical significance of the differences.

**Table 6:** Sexual functions, sexual quality of life, and mood level in the fertile women with type 2 diabetes and the controls during the follicular or luteal phases of the menstrual cycle.

Parametr		Type 2 Diabetes			Controls		
		Follicular Phase (Group IIA) (*n* = 39)	Luteal Phase (Group IIB) (*n* = 54)	*p*	Follicular Phase (Group CIIA) (*n* = 32)	Luteal Phase (Group CIIB) (*n* = 25)	*p*
**FSFI**	Desire	4.2 (3.6–5.4)	4.8 (3.6–6)	1.00	4.2 (3.6–4.8)	4.2 (3.6–4.8)	1.00
	Arousal	4.8 (3.6–5.7)	4.95 (4.2–6)	0.72	5.1 (4.2–5.4)	5.1 (4.8–5.4)	1.00
	Lubrication	5.4 (4.5–6)	5.4 (4.8–6)	0.97	5.5 (4.8–5.8)	5.4 (4.8–6)	1.00
	Orgasm	4.8 (4.4–5.6)	5.2 (4.8–5.6)	0.97	5.2 (4.4–5.4)	5.2 (4.8–5.6)	0.98
	Satisfaction	4.8 (4.4–6)	4.8 (4.8–6)	1.00	4.8 (4.8–5.2)	5.2 (4.8–5.6)	1.00
	Pain	5.6 (4.8–6)	6 (4.8–6)	0.98	5.6 (4.4–5.8)	5.2 (4.4–6)	1.00
	Total FSFI	28.5 (26.9–33.2)	30.5 (27.8–34.1)	0.91	29.9 (28.3–31.2)	30.9 (29–31.8)	1.00
**SQoL-F**	Factor 1	35 (28–39)	34 (29–39)	1.00	33.5 (30–36)	30 (27–35)	0.86
	Factor 2	22 (18–26)	22 (18–27)	0.98	22 (20–25)	21 (20–25)	0.97
	Factor 3	15 (12–17)	14 (13–17)	1.00	15 (12–16)	14 (10–16)	0.96
	Factor 4	15 (12–17)	14.5 (12–17)	1.00	14.5 (12–16)	15 (12–16)	0.95
	Total SQoL-F	83.5 (74–99)	85 (71–96)	1.00	82 (76.5–90.5)	81 (69–93)	0.96
**BDI (score)**		0 (0–3)	0 (0–3)	0.96	(0–2.5)	0 (0–3)	0.97

Data are medians (first and third quartiles) Notes: *p*-values (Tukey’s test); FSFI—Female Sexual Function Index; SQoL-F—Sexual Quality of Life in Women; Factor 1—psychosexual feelings; Factor 2—sexual and relationship satisfaction; Factor 3—self-worthlessness; Factor 4—sexual repression; BDI—Beck Depression Inventory; *p*—statistical significance of the differences.

**Table 7:** Factors that affect the Female Sexual Function Index (FSFI) depending on the prevalence of type 1 or type 2 diabetes.

	Total FSFI					
	Type 1 Diabetes (*n* = 70)			Type 2 Diabetes (*n* = 93)		
	X_i_	SE	*p*	X_i_	SE	*p*
**Intercept**	21.33	1.28	<0.001	38.86	2.39	<0.001
**Phase of the Menstrual Cycle**	5.95	0.36	<0.001			
**Age**				−0.20	0.06	0.002
**BDI**				−0.44	0.08	<0.001
***R*^2^**	0.429			0.358		
**Standard Error of Estimation**	3.46			3.76		
***p* (ANOVA)**	<0.0001			<0.0001		

Notes: X_i_—directional coefficient of the line; SE—standard error of the directional coefficient of the straight; *R*^2^—values of the coefficients of determination; SQoL-F—Sexual Quality of Life in Women; BDI—Beck Depression Inventory.

**Table 8:** Factors that affect the sexual quality of life-women (SQol-F) depending on the prevalence of type 1 and type 2diabetes.

	Total SQoL-F					
	Type 1 Diabetes (*n* = 70)			Type 2 Diabetes (*n* = 93)		
	X_i_	SE	*p*	X_i_	SE	*p*
**Intercept**	114.05	15.42	<0.001	−3.22	9.11	0.724
**Phase of the Menstrual Cycle**	−21.20	3.35	<0.001			
**Age**	−1.93	0.64	0.004			
**Total FSFI**	1.44	0.37	<0.001	2.56	0.30	<0.001
***R*^2^**	0.389			0.443		
**Standard Error of Estimation**	10.1			13.39		
***p* (ANOVA)**	<0.0001			<0.0001		

Notes: X_i_—directional coefficient of the line; SE—standard error of the directional coefficient of the straight; *R*^2^—values of the coefficients of determination; FSFI—Female Sexual Function Index; BDI—Beck Depression Inventory.

## Data Availability

The data could be obtained from the corresponding author on reasonable request.
